# The DOE-JGI Standard Operating Procedure for the Annotations of Microbial Genomes

**DOI:** 10.4056/sigs.632

**Published:** 2009-07-20

**Authors:** Konstantinos Mavromatis, Natalia N. Ivanova, I-Min A. Chen, Ernest Szeto, Victor M. Markowitz, Nikos C. Kyrpides

**Affiliations:** 1 Genome Biology Program, Department of Energy Joint Genome Institute, Walnut Creek, California, USA; 2 Biological Data Management and Technology Center, Lawrence Berkeley National Laboratory, Berkeley, California, USA

**Keywords:** Joint Genome Institute, gene prediction, functional annotation, GeneMark, Metagene, tRNA-Scan, RNAmmer, Rfam, IMG-ER

## Abstract

The DOE-JGI Microbial Annotation Pipeline (DOE-JGI MAP) supports gene prediction and/or functional annotation of microbial genomes towards comparative analysis with the Integrated Microbial Genome (IMG) system. DOE-JGI MAP annotation is applied on nucleotide sequence datasets included in the IMG-ER (Expert Review) version of IMG via the IMG ER submission site. Users can submit the sequence datasets consisting of one or more contigs in a multi-fasta file. DOE-JGI MAP annotation includes prediction of protein coding and RNA genes, as well as repeats and assignment of product names to these genes.

## Introduction

The DOE-JGI Microbial Annotation Pipeline (DOE-JGI MAP) is an automated pipeline for the annotation of bacterial and archaeal genomes using the Integrated Microbial Genome (IMG) system [1]. Annotation includes both the identification of protein-coding and non-coding genes and repeats, as well as the prediction of the function of each gene and the assignment of a product name. The output of this pipeline is available through IMG-ER, which allows genomic analysis and manual curation in a comparative context of hundreds of genomes.

## Requirements

The DOE-JGI MAP uses as input a multi fasta file containing the nucleotide sequences for annotation. In addition, a user is required to provide additional information regarding the project, namely the locus tag prefix for the predicted genes and the method for protein and gene calling. Functional annotation is also optional and the user needs to select whether it will be applied. The pipeline is implemented in Perl and uses a series of publicly available software applications.

## Procedure

### Gene Prediction

**Figure 1:**
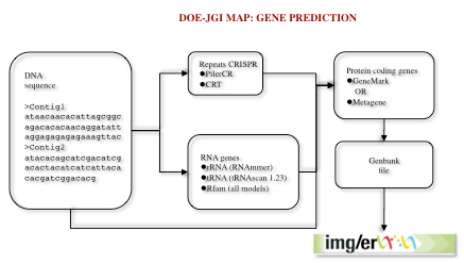
Data flow for gene prediction in the DOE-JGI MAP. Nucleotide sequences are annotated using tools to predict repeats (CRISPR) and RNA genes. Subsequently protein-coding genes are predicted using either GeneMark or Metagene. The consolidated results are then used to create a Genbank file, which is uploaded into the IMG/ER database.

Genes are identified using a combination of Hidden Markov Models and sequence similarity-based approaches. Other features, such as CRISPRs, are also predicted (Figure 1).

The gene prediction pipeline starts with the detection of non-coding RNA genes (tRNA, rRNA and other RNA genes) and CRISPRs, followed by prediction of protein coding genes.

Identification of tRNAs is performed using tRNAScan-SE-1.23 [2]. The domain of the organism (*Bacteria*, *Archaea*) is a parameter that is required; all other parameters are set to default values. Ribosomal RNA genes (5S, 16S, 23S) are predicted using the program RNAmmer [3] using the standard sets of HMMs for RNA genes, provided by the authors. With the exception of tRNA and rRNA, all models from Rfam [4] are used to search the genome sequence. For faster detection, sequences are first compared to a database containing all the ncRNA genes in the Rfam database using BLAST, with a very loose cutoff. Subsequently, sequences that have hits to any genes belonging to an Rfam model are searched using the program INFERNAL, version 1 [5].

Identification of CRISPR elements is performed using the programs CRT [6] and PILERCR [7]. The predictions from both programs are concatenated and, in case of overlapping predictions, the shorter prediction is removed.

Identification of protein-coding genes is performed using either GeneMark (v.2.6r) [8] or Metagene (v. Aug08) [9], both of which are *ab initio* gene prediction programs. The regions identified previously as RNA genes and CRISPRs are masked with Ns in order to avoid prediction of protein-coding genes that overlap RNA genes. GeneMark is run using the parameter “combine”, which combines the GeneMarkS generated (native) and Heuristic model parameters into one integrated model. In the case of draft isolate genomes each contig is treated separately. Metagene is used with its default parameters. At the end of the procedure the masked sequences are replaced with their original content. In the case of an overlap between a protein coding-gene and an RNA gene, the protein-coding gene is truncated to the first start codon (ATG, GTG, TTG) in the same frame that eliminates the overlap or makes it shorter than 30 bp. If this is not possible, the predicted protein-coding gene is removed from the file.

Every annotated gene is given a locus tag of the form PREFIX_#####. Each locus tag is guaranteed to identify a unique gene within this project. However it is up to the user to submit a unique locus tag prefix that will distinguish this project from other genome projects. The number part of each locus tag is a multiple of 10 allowing the future addition of new genes with loci between the existing ones. Loci are simply identifiers and are not guaranteed to have any particular order or internal structure. The output of this stage is a Genbank format genome file, which is uploaded to the IMG-ER database.

### Functional Annotation

**Figure 2:**
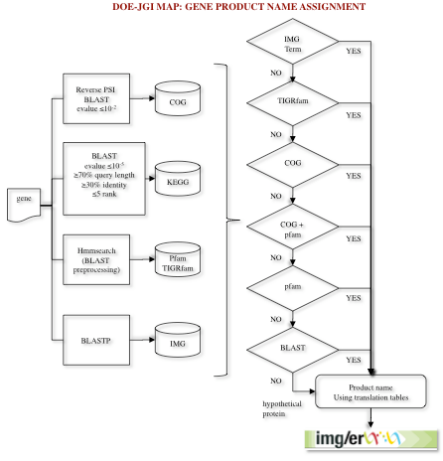
The gene product name assignment procedure used in the DOE-JGI MAP. Genes are first compared to protein families (COGs, Pfam, TIGRfam) and protein databases (KEGG, IMG). A product name is assigned through a series of checks to identify significant hits to IMG terms and the protein families databases. At the end of the process translation tables are used to produce a Genbank compliant product name from the respective source.

After a new genome is processed, the protein-coding genes are compared to protein families (e.g., COGs, Pfam) and the proteome of selected “core” genomes, which are publicly available, and the product name is assigned based on the results of these comparisons (Figure 2). The protein sequences are compared to COG PSSMs obtained from the CDD database [10] using the program RPS-BLAST at an e-value cutoff of 1e-2, with the top hit retained. In addition, the sequences are searched against the KEGG genes database [11] using BLASTp and an e-value cutoff of 1e-5. A KEGG Orthology rank of 5 or better is assigned, with soft masking (-F ‘m S’) and greater than 70% alignment length on the query and KEGG gene sequences. The top hit is retained. Next, the sequences are searched against the Pfam [12] and TIGRfam [13] databases using a BLAST prefiltering and subsequent comparison to HMMs using hmmsearch [14]. The prefiltering is performed by running a BLAST search of the proteome against the seed sequences used to generate an HMM model with an e-value cutoff of 10 and low complexity masking turned off. All hits from the hmmsearch with hit scores better than the per family noise cutoff (--cut_nc) are retained and searched against the IMG proteome database using BLASTp at an e-value cutoff of 10, soft masking (-F ‘m S’) and the top 20 hits are retained.

Our pipeline attempts to assign an IMG term [15] as the gene product name in the first pass; if no IMG term can be assigned, the product name is assigned based on the TIGRfam hit. In the absence of an IMG term and TIGRfam hit, the product name is assigned based on the COG hit or Pfam hit.

Assignment of IMG terms as product names includes the following steps: First, it is verified that the CDS of interest has at least five homologs in the IMG database with >50% identity and at least two of these five homologs have an IMG term assigned. An additional filtering is performed on the homologs with an IMG term based on alignment length in which the alignment length is >70% of the length of both the query and target proteins. A verification that the same combination of IMG terms is assigned to all homologs serves as a consistency check. If all conditions are satisfied, this IMG term (or a combination of IMG terms) is assigned to the CDS of interest as a product name. Multiple IMG terms assigned to the same CDSs are separated by a “/”.

Annotation using TIGRfam hit is attempted, if assignment of an IMG term as a product name fails. If a CDS has a hit above the noise cutoff to only one TIGRfam, the name of this TIGRfam is assigned; if a CDS has hits to more than one TIGRfam, the name of a TIGRfam of the type “equivalog” is assigned. In the case of several hits to TIGRfams of the type “equivalog”, all names of TIGRfams are concatenated into the product name with individual TIGRfam names separated by a “/”.

For the CDSs that were not annotated with either IMG terms or TIGRfam names, the name of the COG hit is assigned as a product name. Under the condition that the CDS has at least 25% identity to the corresponding COG PSSM and the alignment length is at least 70% of the COG PSSM length. If the COG name is “uncharacterized conserved protein” or contains “predicted”, the COG name and COG ID are concatenated in the product name. If either the percent identity or the alignment length condition are not satisfied, the CDSs may still be annotated with this COG name, provided that it has a hit to Pfam, which corresponds to this COG. This condition is verified using the COG-Pfam Correspondence Table. The latter was compiled by mapping Pfams onto COGs through the genes in the IMG database: if all genes in the IMG database with a hit to a certain COG also had hits to the same Pfam (or the same combination of Pfams), this COG and Pfam(s) were designated as “corresponding COG and Pfam”.

For the genes that were not annotated with either IMG terms, TIGRfam or COGs the names of Pfam hits are used as product names. The product name in this case is a concatenation of Pfam family description (attribute “description” in Pfam_family) with “protein”. If a CDS has hits above the noise cutoff to multiple Pfams, their descriptions are concatenated using a “/” as a separator and the word “protein” added in the end.

A translation table for protein product names based on TIGRfam, COG and Pfam descriptions in GenBank is constantly formatted throughout the document. This table has been compiled to make the final product names compatible with GenBank requirements and is used upon submission of the genome to GenBank.

## Implementation

The DOE-JGI MAP is divided in two stages: gene calling and functional annotation. Gene calling and repeat identification is implemented in a series of Perl scripts that call the appropriate software and produce a GenBank file that does not have any functional information for the predicted genes. Subsequently, these genes are loaded into IMG-ER where all the steps of the functional annotation take place. Uploading annotated genomes into IMG-ER and functional annotation occurs in batches every two to three weeks. Submission information is stored in an Oracle database. All programs use default parameters unless stated otherwise in the corresponding section. Updates of databases and software versions occur when a new stable release is available.

## Remarks

The DOE-JGI MAP provides rapid automatic annotation for bacterial and archaeal genomes. It is based on a series of publicly available programs for gene calling and functional annotations. Custom scripts have been developed for the handling of data and integration of different programs. DOE-JGI MAP is a robust pipeline capable of batch annotation for hundreds of genomes in each run. Consistency and reproducibility of the results depend on the databases and software used in the pipeline. New, updated versions of databases like Rfam, Pfam, and KEGG allow the prediction of more genes and more precise annotations. The pipeline is publically available to the genomics community. In order to utilize the DOE-JGI MAP, users need to register and submit their draft or finished genome sequence to the IMG-ER data submission site (http://img.jgi.doe.gov/submit).

It is our intention to keep improving the pipeline by augmenting the existing tools and adding new ones that allow the identification and characterization of more elements in the genomes.
